# New Veterinary Colleges

**Published:** 1881-10

**Authors:** 


New Veterinary Colleges.—We are glad to learn that more veterinary colleges are to be organized in the country. At St. Louis and Philadelphia, efforts are making to establish such institutions, and there seems to be a fair prospect of success. We have now in the United States only two veterinary colleges, both located in this city. Many persons who would like to study veterinary medicine live, perhaps, too far away to do so. On the other hand, a great many persons who have the taste and capacity to make good veterinarians, do not commence the study because its advantages have never been adequately presented.

As a result, the country is flooded with ignorant quacks, a lively and typical picture of whose performances was given by Dr. Navin in our last issue. The two colleges in this city are supplying annually only thirty or forty educated veterinarians.

We believe that there should be more colleges, therefore. We can hardly go so far as Commissioner Loring, who has said that our country needed fifty; but it will be many years before we can fear the establishment of too many.

We must give a word of caution, however, to those who undertake to start a new college. The public still needs to be told that education is essential to a veterinarian. Newly organized institutions will have to work for their patronage. Money will be needed, and a few years must pass before any success can be attained.

Attempts to found veterinary colleges elsewhere than in New York have heretofore been failures. Still, veterinarians are really needed, and so success will surely follow in time intelligent and energetic effort. And we can endorse the belief expressed in the *St. Louis Sportsman*, "that sons of wealthy farmers and storekeepers, in the provinces, will come in numbers to such a college, and the whole district round the home of each graduate will benefit by its institution." It should be borne in mind also that veterinary colleges are very greatly self-supporting, as a charge is made on all animals treated at the hospital.

